# Transient Ischemic Attack-Like Presentation of Extensive Cerebral Venous Thrombosis in a Child With Anaplastic Large Cell Lymphoma

**DOI:** 10.7759/cureus.111574

**Published:** 2026-06-26

**Authors:** Chaymae Cherrabi, Manal Azizi, Amal Bennani, Imane Kamaoui, Ayad Ghanam, Maria Rkain

**Affiliations:** 1 Department of Pediatric Services, Centre Hospitalier Universitaire Mohammed VI, Oujda, MAR; 2 Department of Pediatrics, Mohammed VI University Hospital, Faculty of Medicine and Pharmacy, Mohammed First University, Oujda, MAR; 3 Department of Anatomopathology, Faculty of Medicine and Pharmacy of Oujda, Oujda, MAR; 4 Department of Radiology, Mohammed VI University Hospital, Faculty of Medicine and Pharmacy, Mohammed First University, Oujda, MAR; 5 Department of Pediatric Medicine, Centre Hospitalier Universitaire Mohammed VI, Oujda, MAR; 6 Department of Pediatric Medicine, Faculty of Medicine and Pharmacy, Mohammed First University, Oujda, MAR; 7 Department of Pediatric Gastroenterology, Centre Hospitalier Universitaire Mohammed VI, Oujda, MAR

**Keywords:** anaplastic large cell lymphoma, anticoagulation, cerebral venous sinus thrombosis, pediatric oncology, transient ischemic attack

## Abstract

Cerebral venous sinus thrombosis (CVST) is a rare but serious cerebrovascular complication in children, particularly in the setting of malignancy. We report a case of a 12-year-old boy with anaplastic lymphoma kinase (ALK)-positive anaplastic large cell lymphoma who developed extensive CVST shortly after initiation of chemotherapy. He presented with a transient neurological episode characterized by limb heaviness and dysarthria, initially suggestive of a transient ischemic attack, with complete spontaneous recovery. Although the clinical context raised concern for treatment-related complications, neuroimaging was essential for diagnosis, and magnetic resonance venography confirmed extensive CVST. The patient was treated with anticoagulation using low-molecular-weight heparin, followed by rivaroxaban, with continuation of chemotherapy under close monitoring. Clinical and radiological outcomes were favorable, with complete recanalization after nine months and no neurological sequelae. This case underscores the importance of early recognition of CVST in pediatric oncology patients with atypical neurological symptoms and the pivotal role of prompt neuroimaging.

## Introduction

Cerebral venous sinus thrombosis (CVST) is a rare cerebrovascular disorder in children, characterized by variable and often nonspecific clinical presentation [1]. It is increasingly recognized in pediatric oncology, where hematologic malignancies such as lymphoma represent an important risk setting due to a multifactorial prothrombotic state related to the disease and its treatment. In particular, anaplastic large cell lymphoma (ALCL) may, in some cases, involve or originate from the central nervous system, further increasing diagnostic complexity [2,3]. Clinical manifestations are usually typical, while transient neurological episodes mimicking transient ischemic attacks remain exceptional [4].

## Case presentation

A 12-year-old boy born to non-consanguineous parents presented with a three-month history of asthenia and exercise intolerance, which had initially been neglected. One month later, he developed torticollis during a gymnastics session. Clinical evaluation by a pediatrician revealed bilateral laterocervical lymphadenopathy, for which cervical ultrasound and laboratory investigations were requested but not immediately performed. The patient subsequently developed rapidly progressive exertional dyspnea, prompting referral to our institution.

On admission, he was conscious, asthenic, and hemodynamically and respiratory stable. Physical examination revealed firm right laterocervical lymphadenopathy with obliteration of the right supraclavicular fossa. The remainder of the clinical examination was unremarkable.

Laboratory findings are summarized in Table 1. Chest radiography demonstrated mediastinal widening associated with a right upper parahilar opacity with spiculated margins and central lucency. Chest computed tomography revealed bulky mediastinal, hilar, and supraclavicular lymphadenopathy, some of which were necrotic, associated with multiple pulmonary parenchymal nodules (Figure 1).

**Table 1:** Laboratory findings at admission. Hb: hemoglobin; Hct: hematocrit; MCV: mean corpuscular volume; MCH: mean corpuscular hemoglobin; MCHC: mean corpuscular hemoglobin concentration; PLT: platelets; WBC: white blood cell count; ANC: absolute neutrophil count; LDH: lactate dehydrogenase; AST: aspartate aminotransferase; ALT: alanine aminotransferase.

Parameters	Result	Reference range
Hb	11.9 g/dl	13-18 g/dl
Hct	32.0%	40-54%
MCV	70 fL	80-98 fL
MCH	23.10 pg	27-32 pg
MCHC	32.8%	32-36%
PLT	502000 cells/µl	150,000-400,000cells/µl
WBC	5060 cells/µl	4,000-10,000 cells/µl
ANC	2333 cells/µl	1,500-7,000 cells/µl
Lymphocytes	2039 cells/µl	1,000-4,000 cells/µl
Monocytes	638 cells/µl	200-800 cells/µl
Eosinophiles	60 cells/µl	0-500 cells/µl
Creatinine	6.10 mg/l	5.7-11.1 mg/l
Serum sodium	135 mEq/l	138-145 mEq/l
Serum potassium	4.1 mEq/l	3.4-4.7 mEq/l
Serum calcium	82 mg/l	84-102 mg/l
LDH	437 UI/l	125-243 UI/l
Uric acid	37 mg/l	20-60 mg/l
AST	19 UI/l	5-34 UI/l
ALT	11 UI/l	0-55 UI/l

**Figure 1:**
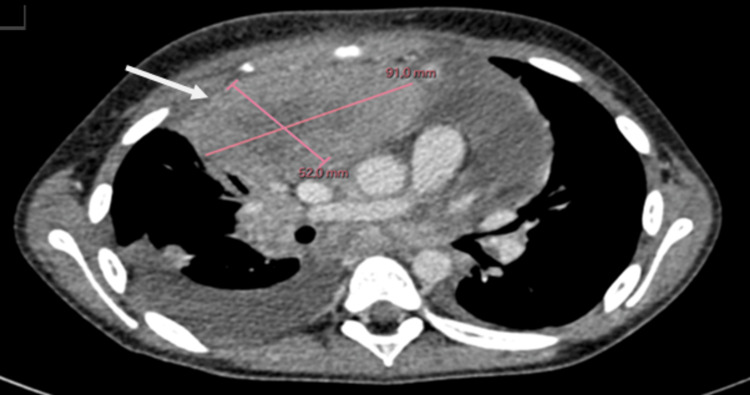
Coronal chest CT showing the extent of mediastinal disease. Contrast-enhanced coronal chest computed tomography (CT) revealing extensive mediastinal involvement with a large mediastinal mass measuring approximately 9.1 cm in its greatest dimension (arrow).

Excisional biopsy of a cervical lymph node confirmed the diagnosis of anaplastic lymphoma kinase (ALK)-positive, CD30-positive ALCL (Figure 2). Staging workup, including contrast-enhanced thoracoabdominal-pelvic computed tomography, showed multiple supradiaphragmatic lymph nodes (Figure 3). Staging investigations, including contrast-enhanced thoracoabdominal-pelvic CT, bilateral bone marrow biopsy, cytospin analysis, and testicular ultrasonography, were unremarkable.

**Figure 2:**
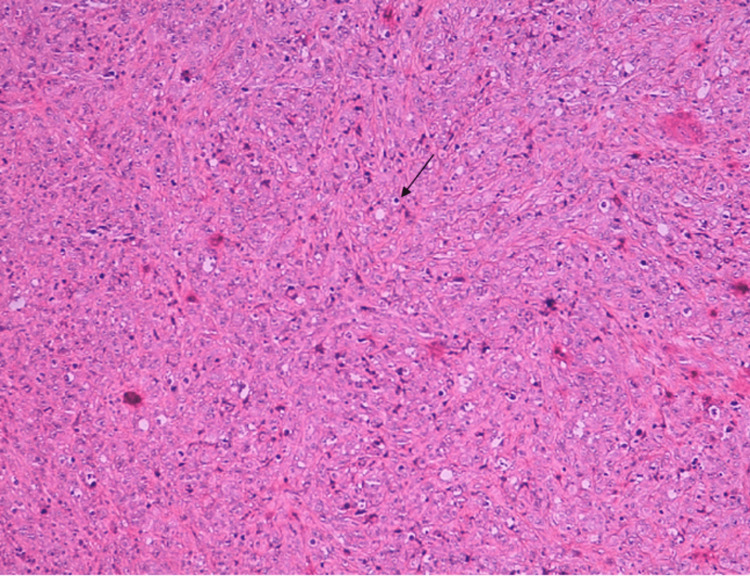
Histopathological examination of the cervical lymph node. Hematoxylin and eosin-stained section showing diffuse infiltration of the lymph node by large atypical pleomorphic lymphoid cells with abundant cytoplasm and irregular nuclei, consistent with anaplastic large cell lymphoma (arrow).

**Figure 3:**
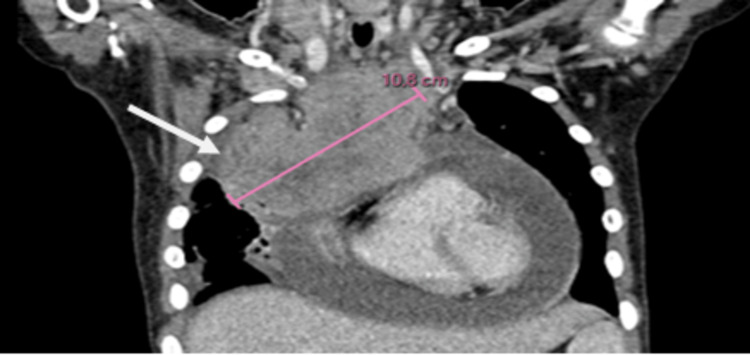
Axial chest CT showing mediastinal involvement. Contrast-enhanced axial chest computed tomography (CT) demonstrating a bulky anterior mediastinal mass associated with extensive mediastinal lymphadenopathy at diagnosis of anaplastic large cell lymphoma (arrow).

The patient was started on chemotherapy according to the ALCL99 protocol. The first course included methotrexate (1 g/m² on day one), ifosfamide (800 mg/m²/day from day one to day five), cytarabine (150 mg/m² every 12 hours on days four and five), etoposide (100 mg/m²/day on days four and five), intrathecal therapy (methotrexate, aracytine, and hydrocortisone), and dexamethasone (10 mg/m² every 12 hours from day one to day five). Treatment was administered without complications.

Approximately one week after the first chemotherapy cycle, the patient developed a sudden episode of heaviness involving both upper limbs and the right lower limb, associated with dysarthria. The episode occurred in an afebrile context, lasted approximately 20 minutes, and resolved spontaneously without loss of consciousness. Upon presentation to the emergency department, the patient was clinically stable and reported only a mild residual sensation of heaviness in both upper limbs, which rapidly resolved. Neurological examination revealed no focal motor or sensory deficits, cranial nerve abnormalities, or altered level of consciousness. Laboratory testing revealed thrombocytopenia (81 × 10⁹/L), while coagulation studies were within normal limits. Thrombophilia workup (including factor V Leiden mutation, protein C, protein S, and antiphospholipid antibodies) showed no abnormalities.

Brain magnetic resonance venography demonstrated extensive CVST involving the superior and inferior sagittal sinuses, the straight sinus, both transverse sinuses, and multiple bilateral frontal cortical veins (Figures 4, 5).

**Figure 4:**
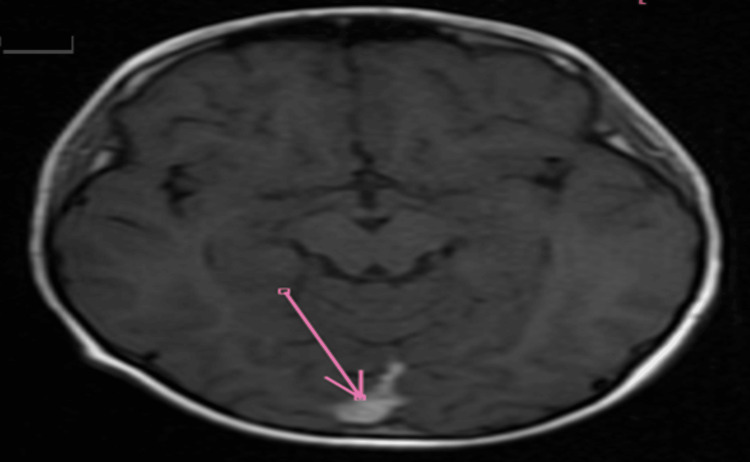
Axial T1-weighted MRI showing thrombus at the torcular Herophili. Axial T1-weighted MRI demonstrating a hyperintense thrombus within the torcular Herophili (arrow), consistent with cerebral venous sinus thrombosis.

**Figure 5:**
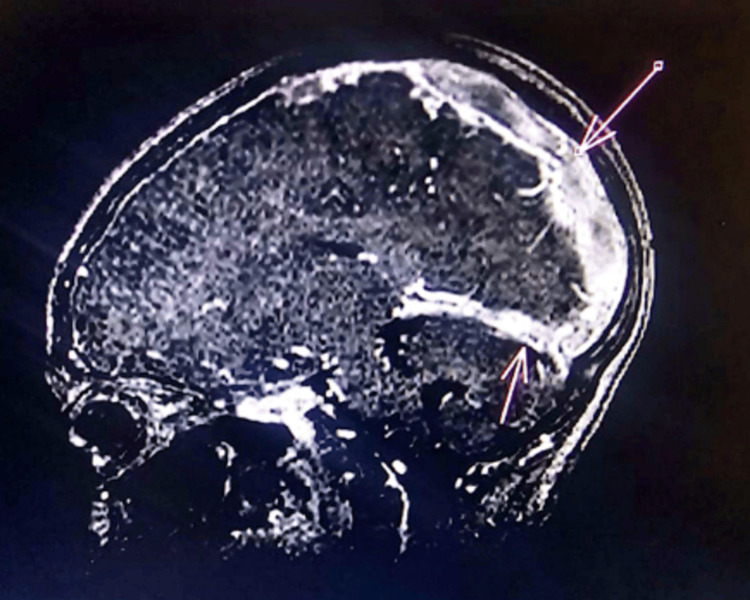
Extensive cerebral venous sinus thrombosis on sagittal brain MRI. Sagittal contrast-enhanced brain MRI demonstrating extensive cerebral venous sinus thrombosis involving the posterior superior sagittal sinus (upper arrow) and extending to the torcular Herophili/transverse sinus region (lower arrow).

Therapeutic anticoagulation with enoxaparin was initiated under close clinical and biological monitoring. After two weeks, treatment was switched to rivaroxaban 15 mg daily. Chemotherapy was subsequently resumed according to the same protocol following hematological recovery.

During follow-up, no recurrent neurological events or hemorrhagic complications were observed. The patient did not experience severe thrombocytopenia requiring interruption or modification of anticoagulant therapy; platelet transfusion support was sufficient during chemotherapy-induced cytopenia. During post-chemotherapy aplasia, he was hospitalized for close clinical monitoring to prevent hemorrhagic complications while continuing anticoagulation under careful surveillance. Clinical evolution was favorable, with progressive improvement in the patient's general condition. Follow-up imaging showed marked regression of the mediastinal mass, leaving a residual anterior mediastinal lesion measuring approximately 20 mm in thickness. Magnetic resonance venography at six months demonstrated persistent thrombosis of the superior sagittal sinus. A subsequent control performed three months later showed complete recanalization of the cerebral venous system with full resolution of thrombosis.

## Discussion

ALCL is a distinct subtype of T-cell non-Hodgkin lymphoma characterized by CD30 expression. The ALK-positive subtype accounts for approximately 95% of pediatric cases and predominantly affects children and adolescents, with a median age at diagnosis of around 12 years. Most patients present with advanced-stage disease, while central nervous system involvement remains uncommon, occurring in only 1% to 3% of cases [3,5].

CVST results from obstruction of cerebral venous outflow, leading to increased venous pressure, cerebral congestion, vasogenic edema, and, in severe cases, venous infarction. Its incidence in children is estimated at approximately 0.67 per 100,000 children. Despite advances in diagnosis and management, this condition remains associated with significant morbidity, with neurological sequelae reported in nearly 38% of patients and a disease-specific mortality of approximately 4% [1,6].

Clinically, CVST is most commonly characterized by headache, altered consciousness, cranial nerve involvement, and/or focal seizures. Presentation as a transient ischemic attack (TIA)-like episode is exceptional and has been reported in only a very limited number of cases [1,2]. In our patient, the neurological episode consisted of transient limb heaviness and dysarthria, initially suggestive of a TIA-like presentation. Although the clinical context in a child undergoing chemotherapy may primarily raise concern for treatment-related complications such as hemorrhagic events or metabolic disturbances, the persistence of a normal neurological examination after symptom resolution warranted further neurovascular evaluation. Magnetic resonance venography ultimately revealed extensive CVST, highlighting the crucial role of early vascular imaging in establishing the diagnosis.

Children with cancer are at increased risk of thromboembolic events due to a complex interaction between the underlying malignancy, treatment-related effects, and, in some cases, an underlying thrombophilic predisposition. Risk factors may be categorized as patient-related and treatment-related factors. Moreover, thrombotic risk varies according to cancer type, with certain hematologic malignancies associated with a higher incidence of thromboembolic complications [2].

In our case, several etiological hypotheses may be considered. First, the underlying disease itself represents a major risk factor for venous thromboembolism. Indeed, lymphoma may promote a prothrombotic state through tumor cell-induced activation of coagulation as well as the production of inflammatory mediators capable of disrupting hemostatic balance [7]. Mediastinal involvement may further increase thrombotic risk, as suggested by a series reporting a 35.7% incidence of thromboembolic events in patients with primary mediastinal large B-cell lymphoma [8]. This increased risk may be explained by venous stasis secondary to compression of adjacent vascular structures by the bulky mediastinal mass. Furthermore, a high tumor burden may further enhance this procoagulant state. Finally, treatment-related factors may also have contributed to thrombogenesis. Methotrexate- and/or doxorubicin-containing regimens, such as Hyper-CVAD (hyperfractionated cyclophosphamide, vincristine, doxorubicin, and dexamethasone), CHOP (cyclophosphamide, doxorubicin, vincristine, and prednisone), and ABVD (doxorubicin, bleomycin, vinblastine, and dacarbazine), have been associated with an increased risk of venous thromboembolism in certain lymphoma series [9]. In our patient, the occurrence of thrombosis one week after the first chemotherapy cycle therefore suggests a synergistic effect between disease-related and treatment-related factors.

From a therapeutic perspective, no specific guidelines exist for the management of CVST occurring in the setting of lymphoma. Cancer-associated venous thromboembolism should therefore be managed according to international recommendations, including those of the International Society on Thrombosis and Haemostasis (ISTH), which recommend low-molecular-weight heparin (LMWH) as first-line therapy in cancer patients with venous thromboembolism, provided creatinine clearance is ≥30 mL/min [7,10].

The minimum recommended duration of anticoagulation in cancer-associated venous thromboembolism is at least six months. Beyond this period, continuation or discontinuation of treatment should be individualized based on periodic reassessment of the risk-benefit balance, taking into account tumor activity, bleeding risk, treatment tolerance, and patient preference. Direct oral anticoagulants represent an effective and safe alternative to LMWH in this setting, with comparable efficacy and no significant difference in major bleeding risk [7,10]. In our patient, anticoagulation was maintained for nine months with rivaroxaban, guided by clinical evolution and imaging findings. The treatment was well tolerated, with no hemorrhagic complications or recurrent thrombotic events, and complete radiological recanalization of CVST was achieved.

## Conclusions

CVST is a rare but potentially serious complication in children with lymphoma. Its clinical presentation may be atypical and, as illustrated in our case, can mimic a TIA despite extensive venous involvement on imaging. This observation highlights the importance of maintaining a high index of suspicion and performing early neurovascular imaging in pediatric oncology patients presenting with acute neurological symptoms. Prompt anticoagulation, together with continuation of oncologic treatment under close monitoring, can result in favorable clinical and radiological outcomes.
